# Thermographic Evaluation of the Stifle Region in Dogs with a Rupture of the Cranial Cruciate Ligament

**DOI:** 10.3390/ani15152317

**Published:** 2025-08-07

**Authors:** Tudor Căsălean, Cristian Zaha, Larisa Schuszler, Roxana Dascălu, Bogdan Sicoe, Răzvan Cojocaru, Andrei Călugărița, Ciprian Rujescu, Janos Degi, Romeo Teodor Cristina

**Affiliations:** 1 Department of Surgery, Faculty of Veterinary Medicine, University of Life Sciences “King Michael I”, 300645 Timisoara, Romania; tudor-mihai.casalean.fmv@usvt.ro (T.C.); larisaschuszler@usvt.ro (L.S.); roxanadascalu@usvt.ro (R.D.); bogdan.sicoe@usvt.ro (B.S.); razvan.cojocaru@usvt.ro (R.C.); george-andrei.calugarita.fmv@usvt.ro (A.C.); 2 Management and Rural Development Department, Faculty of Management and Rural Tourism, University of Life Sciences “King Michael I”, 300645 Timisoara, Romania; rujescu@usvt.ro; 3 Department of Infectious Diseases and Preventive Medicine, University of Life Science “King Michael I”, 300645 Timisoara, Romania; janosdegi@usvt.ro; 4 Department of Pharmacy and Pharmacology, University of Life Science “King Michael I”, 300645 Timisoara, Romania; romeocristina@usvt.ro

**Keywords:** thermal patter, stifle instability, lameness

## Abstract

Background: Canine cranial cruciate ligament (CCL) rupture is a common orthopedic condition leading to stifle joint dysfunction, discomfort, and reduced mobility. Diagnosis typically involves radiography, computed tomography (CT), and magnetic resonance imaging (MRI). In this study, we conducted a retrospective analysis to evaluate the use of infrared thermography in assessing local temperature and thermal patterns in dogs with acute-onset lameness due to CCL rupture compared to those with intact ligaments. Methods: The study involved 12 dogs with cranial cruciate ligament rupture and nine dogs with intact ligaments. The stifle area of all dogs was clipped and scanned using a FLIR E50 thermographic camera. Two regions of interest (ROI), designated El1 and Bx1, were analyzed with FLIR Tools software 5.X by comparing the average of the maximum and of the mean temperature values between the two groups. Results: Thermal imaging revealed differences between the two groups of dogs, which were further supported by significantly higher temperatures in the El1 (lateral aspect of the stifle joint) and Bx1 (cranial aspect of the stifle joint) areas in the study group compared to the control group using a comparative analysis—two-sample *t*-test. In the El1 area, the study group showed a temperature increase of 1.8 °C compared to the control group, while in the Bx1 area, the difference was 1.76 °C. Conclusions: Infrared thermography shows potential to differentiate dogs with acute-onset lameness due to CCL rupture from dogs with intact ligaments, but further studies are needed to assess its accuracy in distinguishing it from other stifle pathologies.

## Simple Summary

Cranial cruciate ligament (CCL) rupture is a common cause of stifle problems in dogs. This study utilized infrared thermography as a diagnostic tool for CCL rupture in dog. Using a thermal camera, we found significantly higher temperatures in dogs with acute onset-lameness due to CCL rupture. Therefore, the results suggest that thermography can help identify CCL ruptures, but further studies are needed to confirm its usefulness in diagnosing other stifle pathologies, such as meniscal tears, collateral ligament injuries, and arthrosis.

## 1. Introduction

The cranial cruciate ligament (CCL) is an essential anatomical structure responsible for provide stability to the stifle joint [1]. It functions by restricting excessive cranial displacement of the tibia relative to the femur [1,2]. Canine cranial cruciate ligament disease (CCLD) is a prevalent orthopedic condition in dogs that causes disfunction, pain, and decreased mobility of the joint [3]. This condition is primarily associated with progressive and degenerative changes within the ligament structure, which significantly contribute to its predisposition to rupture [3]. Several factors contribute to the development of cranial cruciate ligament disease, including aging, conformational deviations like straight pelvic limb alignment, immune-mediated pathologies, hereditary predisposition, excessive biomechanical loading, and reduced vascular perfusion to the mid-substance of the ligament [4,5]. Recent studies suggest that the expression of relaxin and its cognate receptors, LGR7 and LGR8, plays a contributory role in the degradation of ligamentous collagen, thereby representing a potential factor in CCL rupture [6].

Acute cranial cruciate ligament rupture is more frequently observed in dogs under four years of age and is typically the result of trauma-related events [7,8]. Such injuries commonly occur during high-impact activities, including running, jumping, or abrupt changes in direction, where extreme mechanical forces are transmitted through the stifle joint [9]. Symptomatic manifestations of CCL rupture, including sudden onset of severe lameness, joint pain, and functional impairment, generally present immediately following the inciting incident [10,11]. Assessment for craniocaudal laxity of the stifle has become a pathognomonic indicator of CCL injury. Cruciate ligament injury is typically assessed through physical examination, although in some dogs, diagnosing using the cranial drawer test or tibial compression test can be challenging by palpation [3,12]. Both tests reveal increased mobility of the tibia in relation to the femur, indicating instability of the knee joint [1,3,13].

For an accurate diagnosis, the radiographic view requires the stifle to be positioned in 90° of flexion, with hand-applied flexion force to the tarsal area [3]. Flexion of the hock joint facilitates cranial displacement of the tibia, enabling its assessment during this stress view [11]. Radiographic signs include joint swelling, anterior movement of the tibia on the mediolateral projection during tarsal flexion and, in chronic cases, with prolonged conditions [12,13]. Dogs with suspected cranial cruciate ligament dysfunction are typically radiographed to assess for osteoarthrosis, joint effusion, or capsular thickening; however, these outcomes are often nonspecific.

Magnetic resonance imaging (MRI) is the method of choice in human medicine for evaluating stifle disorders [14]. In veterinary practice, MRI arthrography was performed in a series of 11 military working dogs and successfully identified various abnormalities involving the cruciate ligaments, menisci, and collateral ligaments [8,14]. Arthroscopy is a widely utilized modality for the diagnosis and management of CCL ruptures and meniscal tears. However, in comparison to the afore mentioned diagnostic techniques, it is considered to be more invasive [15]. Another diagnostic method is represented by the computed tomography (CT) that become an increasingly employed diagnostic modality for knee pathologies, demonstrating high sensitivity in the identification of teno-ligamentous tears or ruptures, particularly when utilized in conjunction with arthrographic techniques [8]. Stifle ultrasound, a non-invasive diagnostic method, has been studied since the second half of the 1990s [1]. With the continuous advancement of innovative technologies, it has made significant progress in the identification and assessment of primary intra-articular structures like synovitis, osteophytes, osteocartilaginous, and meniscal lesions [16].

Infrared thermography (IRT) is a non-invasive technology that detects infrared radiation emitted by the animal’s body heat and has become an increasingly popular tool in veterinary medicine [17,18,19,20]. This method provides real-time assessment of the skin’s superficial microcirculation [20]. As a highly dynamic organ, the skin continuously adjusts its temperature in response to metabolic processes, facilitating a homeostatic balance between the body’s internal and external temperatures through mechanisms of vasoconstriction and vasodilation [20]. Elevated thermal regions are commonly associated with enhanced local metabolism and blood flow, which may be clinically indicative of an underlying inflammatory process. Conversely, regions exhibiting reduced thermal activity may suggest compromised tissue perfusion, potentially reflecting pathophysiological conditions such as heart failure or disturbances in the nervous system [21,22].

In a study performed by Casas-Alvarado et al., 2024 [20], the mean temperature recorded on the cranio-lateral side of the knee was 31. 2 °C for healthy dogs, compared with a mean temperature of 38.9° C recorded in the same area for dogs with rupture of the cranial cruciate ligament. In a study conducted by Infernuso et al., 2010 [23], the cranial view of intact knee demonstrated a stable and cooler thermal pattern, with the patella presenting as blue, and adjacent to the patella the regions ranged from blue to green. Conversely, in stifles with CCL rupture, the patella retained the same blue thermal pattern, but the parapatellar region exhibited a significantly warmer thermal pattern, transitioning from yellow to orange to red in the cranial view [24]. Similarly, the caudal, lateral, and medial regions exhibited a thermal pattern that was elevated in temperature over bony structures, ranging from yellow to orange [24]. Similar results were observed in a study conducted by Igna et al., 2017 [24], where thermography facilitated the differentiation of deficient stifles from healthy ones based on the distribution of thermal patterns. Specifically, stifles with deficits exhibited thermal patterns of orange and red on the cranial view, while healthy stifles displayed blue to yellow patterns on the lateral side [23].

Thermography can be a valuable method for the evaluation of body surface temperature changes in response to exercise [20,25], for diagnosing pathologies like intervertebral disk disease or rupture of the cranial cruciate ligament [26], and for monitoring how an organism’s body reacts body to drug therapy [27,28].

We hypothesize that dogs with acute-onset lameness due to cranial cruciate ligament rupture will not exhibit notable changes in the local temperature and thermal pattern of the knee area, characterized by an elevation in temperature and alterations in the thermal distribution.

As a physiological response to the local increase in temperature resulting from inflammation associated with cranial cruciate ligament rupture, the objective of the study was to identify temperature differences and thermal patterns between healthy dogs and those with acute-onset lameness due to cranial cruciate ligament rupture.

## 2. Materials and Methods

### 2.1. Animal Assessment and Clinical Examination

The study was conducted at the Surgery Clinic of the Faculty of Veterinary Medicine of Timisoara throughout July 2023 and February 2025.

The inclusion criteria for the study were presentation for orthopedic examination within the first 24 h after the appearance of clinical sign, body weight between 10 kg and 25 kg, acute trauma with severe pain, a positive reaction for tibial compression test and drawer test, and a body temperature between 38 and 39.5° C. The exclusion criteria were defined as patellar luxation in the contralateral limb, joint arthrosis and atrophy of the quadriceps muscle in the affected limb, and prior administration of anti-inflammatory treatment from the onset of clinical signs. For comparison, a control group of nine dogs (Bichon, Dachshund, Cocker Spaniel, and mixed breeds) underwent the same clinical and orthopedic examinations. Inclusion criteria were no administration of anti-inflammatory drugs, body weight between 10 and 25 kg, no lameness in the past two weeks, absence of stifle joint osteoarthritis, and a body temperature between 38.0 °C and 39.5 °C.

From a total of 24 dogs with cranial cruciate ligament rupture that were examined only 12 dogs were included in the study. The dog breeds included in the study were French Bulldog (*n* = 2), Labrador Retriever (*n* = 3), American Staffordshire Terrier (*n* = 2), and mixed breed dogs (*n* = 5). The age of the examined dogs ranged from 3.6 to 7.8 years old (mean: 4.9 years) and the median body weight ranged from 12.1 to 24.9 kg (mean: 18.5 Kg). There were seven female dogs (4 spayed and 3 intact) and five male dogs (3 neutered and 2 intact). Control dogs (*n* = 9) were healthy without any signs of neurologic or orthopedic disease. In the control group, there were 6 female dogs, of which 2 were spayed, and 3 male dogs, of which 1 were neutered.

A clinical and an orthopedic examination was performed by two veterinarians with background in musculoskeletal conditions, who were aware of each other’s results. Dogs were scored for lameness using a scale 0–6 were as follows: 0 = clinically sound/no lameness, walks normally; 1 = mild or slight weight bearing lameness; 2 = obvious or moderate weight bearing lameness; 3 = severe weight bearing lameness; 4 = intermittent non-weight bearing lameness; and 5 = continuous non-weight bearing lameness [24]. The orthopedic examination consists of tibial compression test and drawer test to check the stability of the stifle joint. Both procedures were performed on non-sedated patients.

The cranial drawer test involves grasping the stifle with both hands: the right hand holds the proximal tibia with the index finger on the tibial tuberosity, and the left hand holds the distal femur with the index finger on the patella [29,30]. The tibia is gently pushed forward relative to the femur. No movement indicates a stable stifle. However, in dogs with cranial cruciate ligament instability, there will be a noticeable anterior movement of the proximal tibia compared to the distal femur, often with an indistinct end point [29,30].

The tibial compression test is performed with the patient positioned in lateral recumbency. When the dog is placed on its right side, the examiner’s right hand should cup the stifle, with the index finger extended distally to apply pressure to the tibial tuberosity [29,30]. A cranial displacement of the tibial tuberosity compared to the femur during this maneuver is indicative of cranial cruciate ligament insufficiency or rupture [29,30].

### 2.2. Infrared Imaging and Data Collection

After the medical assessment, the fur surrounding the stifle was removed using a grooming clipper for both groups of dogs.

**Figure 1:**
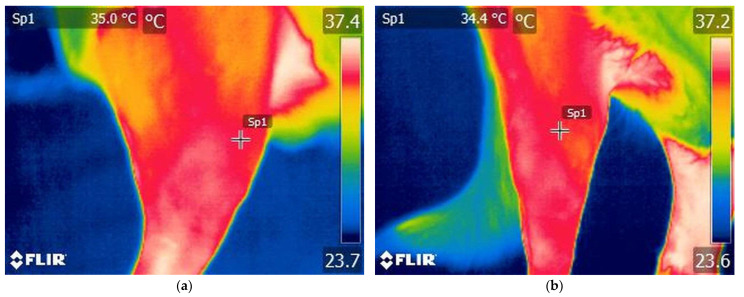
Thermographic image of the stifle joint from a dog with an intact CCL: (**a**) lateral aspect of the stifle, Sp1—spot of temperature; (**b**) cranial aspect of the stifle, Sp1—spot of temperature.

Thermographic examinations were conducted under the same conditions for both groups. The animals were allowed a 30 min acclimatization period in an environment with an ambient temperature of 20–21 °C, with humidity levels between 70% and 75%, and without any noticeable airflow. The operator was placed 1 m from the animal at a 90° orientation to the stifle joint. Following the acclimatization period, the dogs were placed in lateral recumbency with the stifle joint extended, and measurements were recorded from the lateral aspect of the stifle (Figure 1a). Subsequently, the dogs were placed in dorsal recumbency with the stifle joint still extended, and a thermographic image was captured of the cranial region of the stifle (Figure 1b). In every instance, a circular region labeled El1, measuring 33 × 33 pixels, was positioned laterally on the stifle joint, while a second circular region, Bx1, measuring 16 × 33 pixels, was positioned cranially on the stifle joint.

Thermographic imaging was conducted by the same non-blinded operator for both groups of dogs. Thermographic images of the dogs from the control group were acquired between 8 a.m. and 2 p.m., after the clinical examination, but before the orthopedic and radiographic check-up. For the control group, thermographic measurements, orthopedic evaluation, and imaging were performed on the same day. For the study group, images were also collected within the same time frame, depending on the scheduled clinical examination, before orthopedic and radiographic examen.

Thermographic measurements were performed on both groups, and the following categories were established: the control group, taking into consideration the right intact limbs, and the study group with the stifle that presents the rupture of the cranial cruciate ligament.

Thermographic images were captured with the FLIR E50 thermography device (FLIR Systems Inc., Wilsonville, OR, USA) using the following settings: an emissivity value of 0.95 and a resolution of 240 × 180 pixels per image. The temperature range was configured between −20 °C and 650 °C, with a sensitivity of ≤0.05 °C.

The acquired thermographic images were examined and analyzed by the same operator using FLIR Tools software 5.X.

### 2.3. Radiographic Investigation

Radiographic evaluation was performed using two standard views to diagnose CCL rupture [1,3,9,13]. First, a mediolateral projection of the stifle was taken under stress condition to assess joint stability. Second, a craniocaudal view of the stifle was obtained to evaluate joint morphology and alignment [1,9]. Radiographs were analyzed by experienced clinicians and veterinarians, focusing on joint instability indicators, changes in joint space, signs of chronic osteoarthritic alterations, and any cranial displacement of the tibia relative to the femur [9,13].

For radiographic examination with the Siemens Multix Swing device (Munich, Germany), anesthesia was initiated using a combination of medetomidine at 0.03 mg/kg (Domitor 2%, Montero, București, Romania) and ketamine at 4 mg/kg (Ketamidor 100 mg/mL, Richter Pharma, Wels, Austria). If necessary, propofol was administered as a supplementary anesthetic at 5 mg/kg (Propofol 10 mg/mL, Braun, Berlin, Germany).

### 2.4. Statistical Analyses

A total of 21 dogs were subjected to a comparative analysis of values derived from thermographic scanning, comprising a control group (n = 9) and a study group (n = 12). For each thermographic image, the average of the maximum and mean temperature values recorded within the circular regions in FLIR Tools software 5.X was considered. Comparisons focused on temperature differences between the study and control groups, based on data obtained from the El1 and Bx1 areas.

To compare temperature values, we used the two-sample *t*-test. The theoretical formulation of the null hypothesis for each individual comparison is as follows (H0): there are no significant differences between the two studied groups in terms of recorded temperatures. The formulation of the alternative hypothesis (H1) is as follows: there are significant differences. The chosen significance level is α = 0.05. *p*-values below this threshold allow us to reject the null hypothesis in favor of the alternative. Where the exact *p*-values were extremely low—below the detection limit of the software application—the expression “*p* < 0.0001” was used.

Given the small sample sizes, we also conducted a power analysis for the two-sample *t*-test, using the SAS Studio—Power and Sample Size functions. To further validate the comparative results, we additionally applied the non-parametric equivalent—the Wilcoxon rank-sum test.

The software application used for the statistical analysis was SAS Studio version 3.8.

## 3. Results

From twenty-four dogs examined in twenty-one months, twelve were included in the study which met the criteria for inclusion.

The interval between the onset of lameness and clinical presentation ranged from 4 to 24 h, with varying score of lameness (Table 1). All dogs were positive to both the drawer cranial test (Video S1) and tibial compression test (Video S2). Stifle joint swelling was observed in eight of the twelve dogs examined.

**Figure 2:**
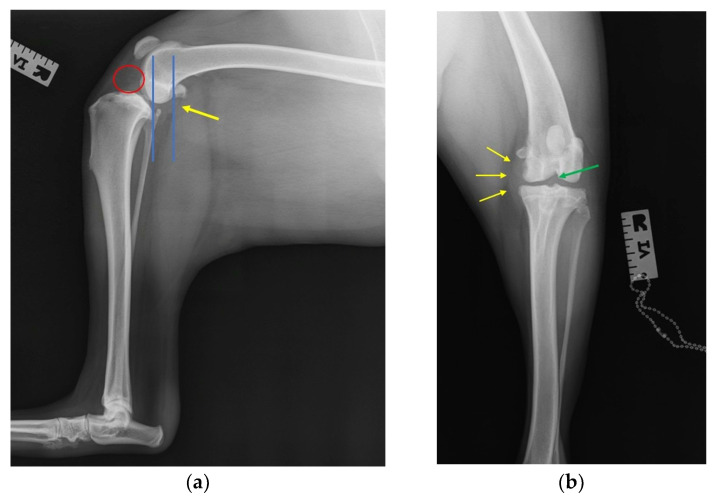
Radiography of the stifle joint in a dog with a rupture of the cranial cruciate ligament: (**a**): mediolateral view, tibial compression test with stifle and tarsus are flexed at 90°; red circle—increased soft tissue radiopacity within the joint space, resulting in cranial translation of the infrapatellar fat pad; blue line—cranial translation of the tibia with tibial eminences is displaced cranially relative to the normal neutral position; yellow arrow—caudal edge of the femoral condyle (**b**) craniocaudal view: yellow arrows—thickening of the medial segment of the joint capsule; green arrow—points to joint effusion.

Radiographs of the dogs included in the study revealed signs of joint instability as displacement of the tibia relative to the femur in all twelve dogs and joint effusion was identified for eight dogs (Figure 2a,b). Also, patellar luxation was associated with CCL rupture in four of the twelve dogs.

### 3.1. Thermography Result

#### 3.1.1. Thermography Scan of the Control Group

**Figure 3:**
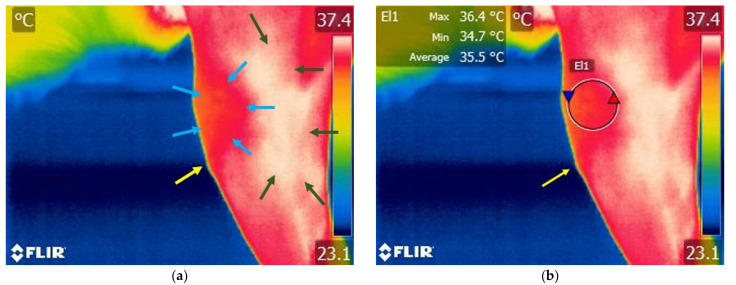
Thermographic image of the left knee in a dog with an intact cranial cruciate ligament—lateral view of the joint. (**a**) Image without Flir Tools software analysis: blue arrows—variation areas of low temperature; green arrows—variation areas of increased temperature; yellow arrow—proximal aspect of the tibial crest tuberosity. (**b**) Scanning image with FLIR Tools software analysis: yellow arrow—proximal aspect of the tibial crest tuberosity; El1—area of interest centered on the knee joint; red triangle spot—maximum temperature recorded in the interest area; blue triangle spot—minimum temperature recorded in the interest area.

**Figure 4:**
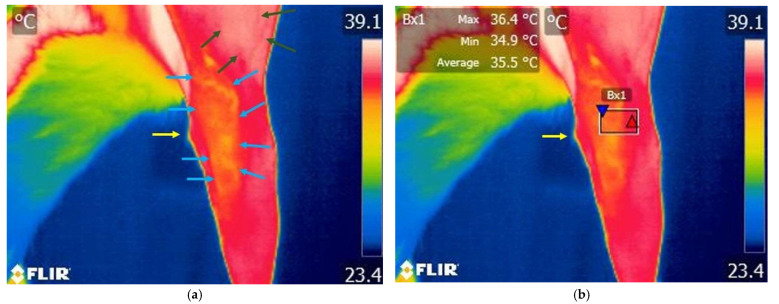
Thermographic image of the left knee in a dog with an intact cranial cruciate ligament—cranial view of the joint; (**a**) image without Flir Tools software analysis: blue arrows—variation areas of decreased temperature; green arrows—variation areas of increased temperature; yellow arrow—proximal aspect of the tibial crest tuberosity. (**b**) Scanning image with FLIR Tools software analysis: yellow arrow—proximal aspect of the tibial crest tuberosity; Bx1—area of interest centered on the knee joint; red triangle spot—maximum temperature captured in the interest area; blue triangle spot—minimum temperature recorded in the interest area.

Various temperature differences could be observed on the lateral side of the knee region. Specifically, lower temperatures are recorded in the cranial aspect of the knee joint, while higher temperatures are present caudally and in the tibial region. Additionally, on the cranial surface of the knee joint, temperature variations remain within lower value ranges (Figure 3 and Figure 4).

#### 3.1.2. Thermography Scan of the Study Group

**Figure 5:**
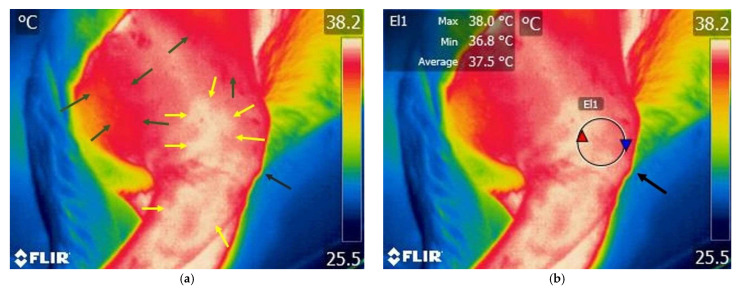
Thermographic image of the right knee lateral area of a dog with a rupture of the cranial cruciate ligament. (**a**) Image without Flir Tools software analysis: green arrows—variation areas of low temperature; yellow arrows—variation areas of increased temperature; black arrow—tibial crest tuberosity. (**b**) Thermal image with FLIR Tools software assessed: black arrow—tibial crest tuberosity; El1—area of interest centered on the knee joint; red triangle spot—maximum temperature captured in the target area; blue triangle spot—minimum temperature captured in the target area.

**Figure 6:**
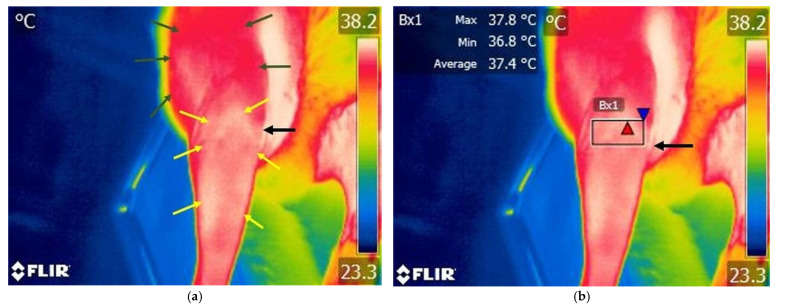
Thermal image of a dog with a rupture of the cranial cruciate ligament—cranial view of the right knee joint. (**a**) Image without Flir Tools software analysis: green arrows—variation areas of decreased temperature; yellow arrows—variation areas of increased temperature; black arrow—tibial crest tuberosity. (**b**) Scanning image with FLIR Tools software analysis: black arrow—tibial crest tuberosity; Bx1—area of interest centered on the knee joint; red triangle spot—maximum temperature captured in the target area; blue triangle spot—minimum temperature captured in the target area; black arrow—tibial crest tuberosity.

Different color variations can be observed, with a predominance of white indicating increased temperature in the lateral region of the knee and distally towards the tibia. Proximally, cranially, and caudally to the femur region, a red area appears, indicating a moderate temperature. Increased area of temperature was identified on the cranial aspect of the knee joint (Figure 5 and Figure 6).

### 3.2. Comparison Within El1 Area

#### 3.2.1. Comparisons for Maximum Temperature

**Figure 7:**
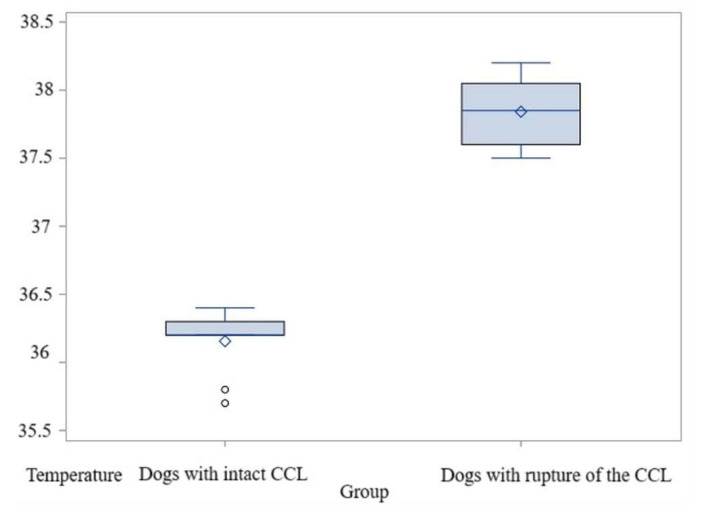
Comparative boxplot indicating to the results obtained for El1 between the dogs with an intact CCL and those with a rupture of the CCL; distribution of the maximum temperatures; ◦—outliers; ◊—mean referee value.

The average temperature was 36.15 °C in the control group, while in the study group the temperature was 37.84 °C. The value of the *t*-test was t = −15.56 and *p* < 0.0001; therefore, the null hypothesis (H0) is rejected. The difference between means is 1.68 °C, with a standard deviation of 0.24. The power of the test is high, exceeding 0.99. The non-parametric analysis likewise revealed statistically significant distinctions between the groups, *p* = 0.001 (Figure S1). Therefore, for the maximum temperatures in the El1 area, the comparisons indicated significantly higher values in dogs with acute-onset lameness due to CCL ruptures than in those with an intact CCL (Figure 7, Table S1).

#### 3.2.2. Comparisons for Average Temperature

**Figure 8:**
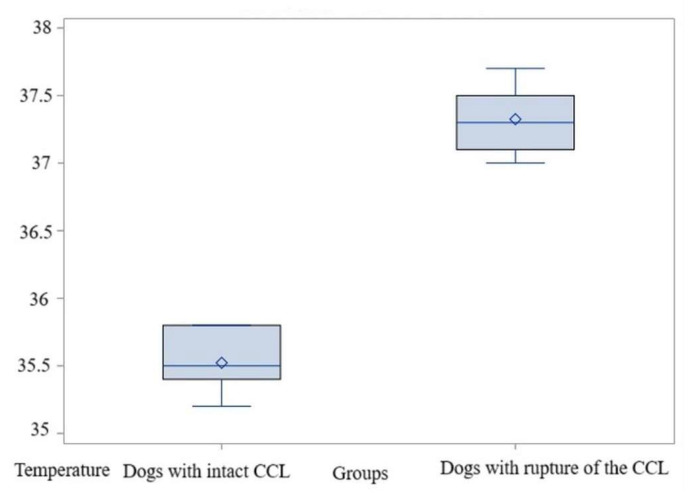
Comparative boxplot indicating the results obtained for El1 between the dogs with an intact CCL and those with a rupture of the CCL; distribution of the average temperatures; ◊—mean referee value.

The average of these values was 35.52 °C in the control group, while in the study group the temperature was significantly higher at 37.32 °C, with a *t*-test value of t = −17.16 and *p* < 0.0001; thus, H0 is rejected. The difference between means is substantial—1.8 °C—with a standard deviation of 0.23. In this case as well, the test power is high, exceeding 0.99. The non-parametric test also indicated significant differences between the groups, *p* = 0.001 (Figure S2). Thus, in the evaluation of the El1 area where the average temperatures were determined, the comparisons also indicated significantly higher values in dogs with acute-onset lameness due to CCL ruptures than in those with intact CCL (Figure 8, Table S1).

### 3.3. Comparison Within Bx1 Area

#### 3.3.1. Comparisons for Maximum Temperature

**Figure 9:**
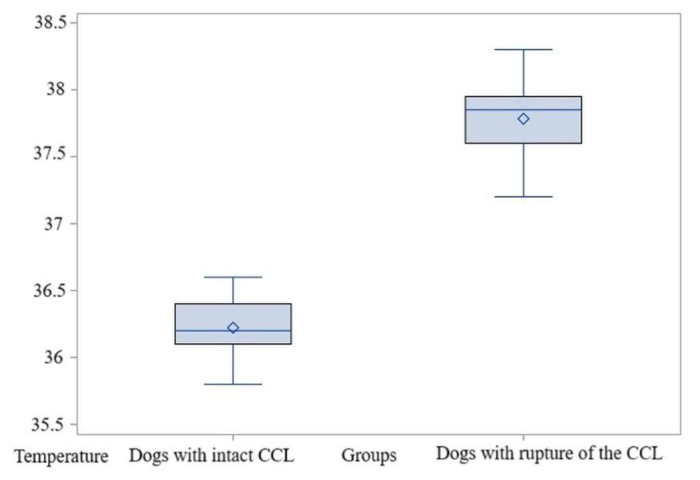
Comparative boxplot indicating to the results obtained for Bx1 between the dogs with an intact CCL and those with a rupture of the CCL; distribution of the maximum temperatures; ◊—mean referee value.

The average of these values was 36.22 °C in the control group, while in the study group the temperature was 37.78 °C, with a *t*-test value of t = −12.63 and *p* < 0.0001. The p-value being lower than the significance threshold α = 0.05 allowed for the rejection of the null hypothesis (H0) in favor of the alternative hypothesis (H1). The difference between means showed a strong contrast relative to the low variability. Specifically, a difference of 1.56 °C was observed between the two groups, while the standard deviation was only 0.28. These values led to an estimated test power greater than 0.99, indicating that the test has a strong ability to detect differences between the groups. The non-parametric Wilcoxon rank-sum test also indicated significant differences between the groups, *p* = 0.001 (Figure S3). Thus, dogs with acute-onset lameness due to CCL ruptures had significantly higher maximum temperatures in the Bx1 area compared to animals with intact CCL (Figure 9, Table S2).

#### 3.3.2. Comparisons for Average Temperature

**Figure 10:**
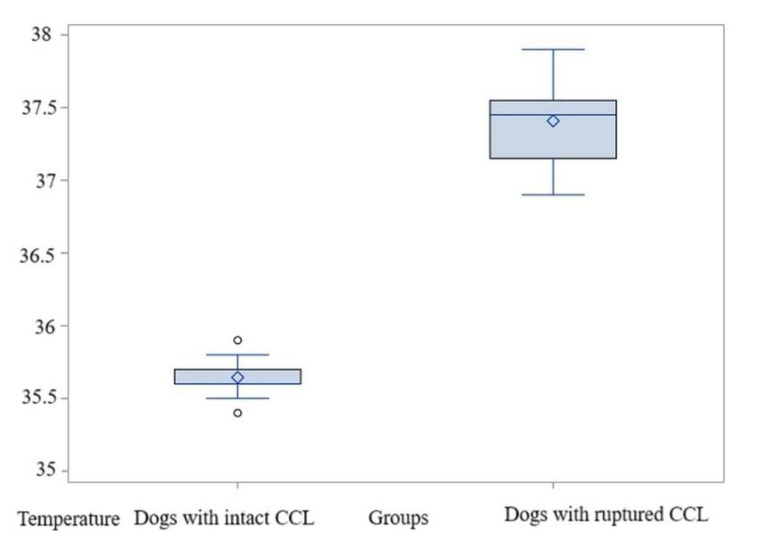
Comparative boxplot referring to the results obtained for Bx1 between the dogs with intact CCLs and those with a rupture of the CCL; distribution of the average temperatures; ◦—outliers; ◊—mean referee value.

In the control group, the average temperature was 35.64 °C, while in the study group it was significantly higher at 37.40 °C. The *t*-test showed a significant difference (t = −13.69, *p* < 0.0001), which allows for the rejection of H0. The large difference between means—1.76 °C—combined with a low standard deviation of 0.24, resulted in a test power of 0.99, indicating high statistical power. The non-parametric test also revealed significant differences between groups (*p* = 0.001). Therefore, animals with acute-onset lameness due to CCL rupture showed significantly higher temperature values compared to those with intact CCL, when evaluating the Bx1 area based on average temperature recordings, and the non-parametric test confirmed this (*p* = 0.001) (Figure 10, Table S2).

## 4. Discussion

Thermal imaging of the knee region yields valuable data for distinguishing dogs with acute-onset lameness due to cranial cruciate ligament ruptures from those with intact ligaments. This is supported by the rejection of the null hypothesis, indicating significant differences in both maximum and average temperatures recorded from the El1 and Bx1 areas in the two groups. The results showed that dogs with acute-onset lameness due to CCL rupture exhibited altered thermal patterns and higher temperature values in the selected areas compared to dogs with intact ligaments.

The principle of thermography relies on the increase in metabolic activity and blood circulation within an affected area [11,16]. Because the demand for nutrients is higher in damaged or inflamed tissue compared to healthy tissue, the local temperature rises. It is well established that inflammation is associated with localized hyperthermia [28]. By using medical thermography, we can gather valuable information about blood flow in specific regions and detect potential inflammatory processes, which may indicate the presence of disease [16].

In our study, for both regions where we analyzed El1 and Bx1, the differences in maximum and average temperatures between dogs with acute-onset lameness due to rupture of the CCL and intact ones exceeded 1 °C (Tables S1 and S2). According to Infernuso et al., 2010 [23], utilized infrared thermography (IRT) to discern between healthy stifles and those afflicted with CCL rupture in a cohort of sixteen dogs. Their results indicated that the mean surface temperature of the affected stifle was elevated by approximately 1 °C compared to the contralateral, unaffected limb [26]. Recent studies have demonstrated that injured stifles exhibit a significantly higher surface temperature compared to healthy stifles, with a localized increase of approximately 2.4 °C to 4.6 °C in the affected joints [31].

In musculoskeletal trauma, local tissue temperature has been reported to increase by approximately 1 to 4 degrees Celsius relative to unaffected regions [32]. This thermal elevation is indicative of the underlying inflammatory processes, characterized by enhanced vascular perfusion and heightened metabolic activity within the affected tissues [33].

Rectal temperature was recorded for each animal to confirm that abnormal body temperatures, such as hypothermia or hyperthermia, would not affect the outcomes. The comparison of mean rectal temperatures revealed no statistically significant differences between the groups. Nomura et al. [34] identified no differences in rectal temperature between intact cranial cruciate ligament and dogs with rupture of the cranial cruciate ligament.

In our study the thermographic scans we performed on both groups of dogs were carried out after trimming the hair from the region of the femur trochanter down to the level of the hock to not influence the local temperature because the fur can play as an insulator [35]. Local temperature can be impacted by the characteristics of the hair, including its presence, color, length, type, and distribution [35]. Authors observed that short-haired animals showed elevated surface temperatures, exceeding those of other animals by about 2 °C [36]. The absence of a dense insulating layer in animals with short hair, compared to those with long or double coats, likely facilitates the more evident increase in IRT values [36].

The dogs benefited from a 30 min acclimation period in a space where the ambient temperature was 21 °C. The same acclimation period and ambient temperature were used in other studies involving the local evaluation of knee temperature [37]. Infernuso et al., 2010 [23], obtained thermographic measurements of the knee joint in a room where ambient temperatures ranged from 20 to 24 °C, representing a greater variability compared to the controlled room temperature maintained in our study.

The diagnostic process includes evaluating lameness history, anamnesis, and particular diagnostic procedures such as radiography and arthroscopy [11,38]. In our study the mean age of the dogs diagnosticated with ruptures of the CCL was 4.9 years, which is slightly younger than the 7–10 years of age previously reported [10]. The dogs included in the study suffered of acute-onset lameness due to CCL rupture during arduous activity or work, risk factors that have also been identified by other authors [39]. Chronic pelvic limb lameness in middle-aged, medium and large breed dogs often leads to an abrupt and severe lameness episode after only mild exercise [1]. This clinical presentation is commonly associated with underlying stifle pathology, including CCL disease [39].

CCL injuries in dogs are generally categorized into two types: a degenerative condition that commonly affects middle-aged to older dogs, and a traumatic condition that tends to occur in younger dogs as a result of traumatic injuries [1,10,40]. In chronic cases, after the ligament has been compromised, joint instability increases significantly, leading to cartilage degeneration, meniscal injury, osteophytes, and progressive osteoarthritis [41,42]. Dogs that present CCL rupture in one knee have a high risk of a following CCL in the contralateral one, or to develop osteoarthritis in both stifles [43]. In our study, we included dogs with trauma of the CCL, cases with a progression of less than 24 h, presenting clinical signs such as acute lameness, stifle pain, and intra-articular effusion.

In our study, we identified CCL rupture in Labrador Retriever, American Staffordshire Terrier, and mixed-breed dogs. Other authors have reported similar findings, noting that the ten most commonly affected breeds were mixed-breed dogs, Labrador Retrievers, Rottweilers, Golden Retrievers, Cocker Spaniels, German Shepherd Dogs, Boxers, Bichon Frise, Siberian Huskies, and Doberman Pinschers [3].

Numerous authors have reported that, during clinical examination, the most consistent findings in cases of stifle joint injury are joint swelling and instability [12,44,45,46,47]. To evaluate this instability, two diagnostic tests are routinely used in veterinary practice: the cranial drawer test and the tibial compression test [11]. In our study, all dogs in the study group tested positive on both the cranial drawer test and the tibial compression test.

To confirm the CCL rupture, authors use radiography of the knee joint using mediolateral and craniocaudal views [13,22]. For subtle instability, stress radiography under sedation can demonstrate tibial translation [1]. Also, patellar luxation may also be identified radiographically and may increase the index of suspicion for CCL rupture [7]. In our study, all twelve dogs present cranial translation of the tibia with respect to the femur and four dogs present medial patellar luxation of the tibia.

Unlike other imaging modalities, thermography is a completely non-invasive technique that requires neither anesthesia nor exposure to ionizing radiation [28,39]. This makes it particularly suitable for use in veterinary medicine, where clinicians often manage uncooperative, fearful, or painful animals [1,8,10,48]. Thermography enables the rapid acquisition of infrared images, which can be immediately visualized and analyzed with minimal patient handling. Numerous studies have highlighted the ability to detect changes in thermal patterns before clinical signs or radiographic abnormalities appear, enabling early identification of pathological processes and facilitating timely clinical intervention [12,21,49,50]. Additionally, once the initial cost of the thermal imaging system is offset, the operational expense per examination remains low, further enhancing its practicality in routine veterinary practice [51].

These findings suggest that dogs with acute-onset lameness due to CCL rupture demonstrate increased local temperatures in the affected stifle joint compared to those with intact ligaments, likely reflecting underlying inflammatory processes and stifle pain. Further research is warranted to expand upon these observations. Specifically, future studies should (1) compare stifle temperatures within the same individual, between the affected joint and the contralateral, clinically unaffected stifle, to control for individual variation; (2) investigate the utility of thermography in distinguishing temperature patterns between stifles with acute CCL rupture and those with osteoarthritic changes; (3) assess local temperature differences among dogs presenting with acute CCL rupture and other stifle pathologies, including patellar luxation, collateral ligament rupture, and meniscal injuries; (4) evaluate whether coat clipping influences thermographic measurements in dogs with CCL rupture; (5) assess local temperature differences among dogs presenting with acute CCL rupture and dogs with septic arthritis or leishmaniosis; and (6) monitor perioperative and postoperative temperature changes following various orthopedic stabilization techniques to explore their impact on local inflammatory responses and tissue recovery.

This study presents several limitations that warrant consideration. Firstly, the inclusion of multiple canine breeds without breed-specific analysis may have introduced confounding factors, given the anatomical, physiological, and thermoregulatory differences inherent to various breeds. Secondly, the decision to clip hair in the evaluated regions may have influenced thermographic measurements, as hair length and density are known to affect surface temperature readings, potentially compromising the accuracy of thermal imaging. Furthermore, the absence of advanced diagnostic modalities such as magnetic resonance imaging (MRI) limited the capacity to definitively diagnose conditions including osteochondrosis, meniscal injuries, and collateral ligament pathologies, which may have impacted the reliability of the clinical assessments. Lastly, the use of a thermographic device with moderate resolution may have restricted the sensitivity and precision of the thermal data obtained, potentially hindering the detection of subtle temperature asymmetries associated with musculoskeletal abnormalities.

## 5. Conclusions

The findings of this study indicate that the local temperature in the stifle area of dogs with a ruptured cranial cruciate ligament is higher compared to the same area in dogs with an intact ligament.

The thermal pattern of the stifle area present differences between dogs with rupture of the cranial cruciate ligament compared with healthy ones.

Additional research is warranted to determine its effectiveness in distinguishing CCL ruptures from other stifle pathologies, including meniscal tears, collateral ligament injuries, leishmaniosis, septic arthritis, and arthrosis.

## Figures and Tables

**Table 1:** Different score of lameness for the digs with ruptures of the CCL.

Score of lameness	2	3	4
Dogs with CCL	7	3	2

## Data Availability

The original contributions presented in the study are included in the article/Supplementary Materials; further inquiries can be directed to the author.

## Supplementary Materials

The following supporting information can be downloaded at https://www.mdpi.com/article/10.3390/ani15152317/s1; Figure S1: Distribution of Wilcoxon score for Maximum in El1; Figure S2: Distribution of Wilcoxon score for Average in El1; Figure S3: Distribution of Wilcoxon score for Maximum in Bx1; Figure S4: Distribution of Wilcoxon score for Average in Bx1; Table S1: Mean value of temperature El1 area; Table S2: Mean value of temperature Bx1 area; Video S1: Positive drawer test in a dog with a rupture of the cranial cruciate ligament; Video S2: Tibial compression test in a dog with a rupture of the cranial cruciate ligament.
