# Supplementary material for: Thermographic Evaluation of the Stifle Region in Dogs with a Rupture of the Cranial Cruciate Ligament
Source: Animals (Basel). 2025 Aug 7;15(15):2317. doi: 10.3390/ani15152317 (PMC12345753; doi:10.3390/ani15152317)
Supplement: Supplementary file 1 [file animals-15-02317-s001.zip › Supplementary materials/Agreement document.pdf]

COLLEGE OF VETERINARY DOCTORS

EXECUTIVE OFFICE

Splaiul Independenței 105, sector 5, Code 050097, BUCHAREST

Phone number/ Fax 319.45.04; 319.45.05 www.cmvro.ro

E-mail: office@cmvro.ro

Operator of personal data no. 21554/2011

Under:

- Law no. 160 of July 30, 1998 for the organization and exercise of the profession of veterinarian; republished with subsequent amendments and completions,
- Regulation of Organization and Functioning of the College of Veterinarians, art. 17 lit. h) and r).

The National Council of the College of Veterinarians adopts:

DECISION NO. 34101.12.2012

regarding the approval of the consent model for the acceptance of the medical procedures / operators and of the model of solicitation / acceptance of euthanasia

Art. 1. The consent model for the acceptance of the medical-operative procedures provided in Annex no. 1 which is part of this decision.

Art. 2. The euthanasia request / request / acceptance model provided in Annex no. 2 which is part of this decision.

Art. 3. (1) This decision shall enter into force starting with the date of Ol - Ol 2013

(2) After the date of entry into force of this Decision, the use of the two forms shall be mandatory for all veterinary care units,

Art. 4. This Decision was adopted by the National Council of the College Veterinarians with unanimous votes, today 01.12.2012.

President

College of Veterinarians

Conf. Univ. Dr. Viorel ANDRONIE

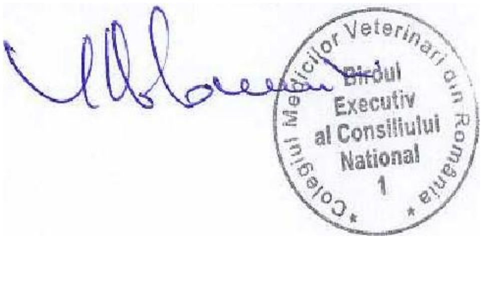

Annex No. I

CONSENT FOR ACCEPTANCE

MEDICAL / OPERATING PROCEDURES-MODEL

Owner / S.C.______________________________________

adress in____

as the owner/ legal representative of the patient, ID card No._____________.
Name of the patient,_____________________, age_____,
breed________________, gender______, ID number__________________,

phone number____________________

I agree wit the following medical/surgical procedure

____

Anesthesia____

Description of the medical/surgical procedure___________________________

____

____

The nature and purpose, benefits and risks of performing / not performing this medical procedure, as well as other therapeutic operations, have been explained to me in detail and on my understanding by Dr. ____________________________

I was informed regarding to the associated risks as well as the unpredictable risks (including the slightest risk of death) of the consequences of the medical procedure, as well as the risks of special investigations that are part of the medical procedure to be borne by the patient I represent.

I declare that I am aware of these risks and accept them, as the purpose of the medical procedure is for the good of the patient who represents him.

As a result, I understand the need for this medical procedure that I want to perform and acknowledge that I cannot be given a guarantee or assurance as to the end result.

If unforeseen situations and conditions arise during the medical / therapeutic / surgical intervention, which require additional procedures to those described above as being accepted by me (including the transfusion), I accept that the doctor will act accordingly. I declare that I agree with the doctor to make any decision in time medical procedure for the good of the patient

In order to perform the medical / surgical procedure, I also consent to the administration of the anesthesia that was indicated to me (general / other type) The risks of the anesthetic act were explained to me and I understood them during the consultation.

Consequently and under the specified conditions, I give my free and informed consent to the medical procedure / operative intervention / treatment [present.

I declare that I will receive all the indications regarding the postoperative care that I have to apply at home and I assume the possible complications caused by the strict non-observance of the indications received.

I certify that I have read, understood and fully accept the above and that I therefore sign them.

Any medical procedure involving general anesthesia of any kind requires the observance of a fast 12 hours before the intervention. I declare on my own responsibility that the patient has not consumed solid food since:
hour________date________________.

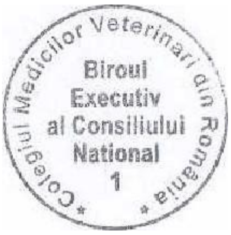

I declare on my own responsibility that at the time of signing this act, I did not consume alcohol, hallucinogenic substances, etc. and I am fully lucid and informed.

It is recommended that the following investigations be performed before performing the medical procedure:____

I declare that I agree /do not agree with biochemical analyzes,

I declare that I agree /do not agree with hematological analyzes,

I declare that I agree /do not agree with ultrasound investigations,

I declare that I agree /do not agree with radiological investigations, MRI ,

CT with or without contrast agent,

I declare that I agree /do not agree with cardiological investigations,

I declare that I agree /do not agree with biochemistry and urinary sediment, I declare that I agree /do not agree with blood ionogram and blood gases, recommended by to the veterinarian, necessary for the medical procedure / operators and I assume all the risks deriving from this refusal: failure of the medical procedure / operation and / or death of the animal or possible postoperatory complications .

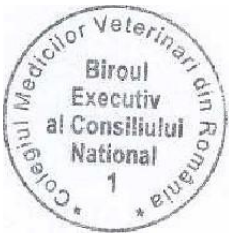

Carrying out the investigations accepted by the owner does not guarantee the success of the medical maneuvers, they only reduce as much as possible the possible complications.

Following violent traumas at the muscular level, by devitalizing the issues, a phenomenon of cell destruction can be installed, resulting in the necrosis of the affected and adjacent tissue.

Acute renal failure can occur in case of severe muscle damage as well as Bywaters Syndrome that occurs as a result of extensive skeletal muscle injuries, caused by compression, crushing or amputation. It is the consequence of the release into the bloodstream of myoglobin. Present in large quantities in the blood, it quickly becomes toxic and blocks the renal tubules, which causes acute renal failure.

In the case of surgeries involving pyothorax, peritonitis, pyometra, prostate abscesses, liver and kidney abscesses, orchiepididymitis, superinfected wounds and tumors, Systemic Inflammatory Response Syndrome (S.I.R.S) may occur postoperatively. - which represents the totality of the body's defense reactions against septic aggression. It is a serious pathology (50% mortality in optimal therapeutic conditions), so the treatment, symptomatic and etiological, must be undertaken as early as possible without guaranteeing the saving of the animal's life.

I declare that I have received instructions on postoperative care at home, as well as the return visit schedule after the medical / surgical procedure.

I hereby give my consent for the start of the treatment / procedure / operation and I agree to pay the value of the treatments, analyzes and medical-surgical interventions that were explained and understood by Dr____

from the Veterinary Medical Office

They were presented to me and I took note of the estimated costs between the amount of: __________ and the amount of:_________

I certify that I have read, understood and fully accept the above and as a result I sign_____________________________

Signature of the legal representative / Owner day month year The undersigned.

___________________ day______month_________ year_______

I confirm that this consent form was completed in my presence and signed by the owner without any coercion

Witness signature ____________ day________ month_______ year ________

* A copy of the identity document is attached
