# Supplementary material for: Thermographic Evaluation of the Stifle Region in Dogs with a Rupture of the Cranial Cruciate Ligament
Source: Animals (Basel). 2025 Aug 7;15(15):2317. doi: 10.3390/ani15152317 (PMC12345753; doi:10.3390/ani15152317)
Supplement: Supplementary file 1 [file animals-15-02317-s001.zip › Supplementary materials/Consent for Cooperation.pdf]

Number. ..../..................
U.L.S. “Regele Mihai I” from Romania - F.M.V. – University Veterinary Clinics

Cooperation Consent

Between:

University of Life Sciences “Regele Mihai I” from Romania, – Faculty of Veterinary Medicine Timisoara (F.M.V.), represented by Prof. univ.dr. Morariu Sorin, as dean

And

A) **Individual person/S.C. ....** with address in ...., like owner / legal representative, legitimized with

B.I./C.I series ...... Number............................,issued by..................................at date ...., CNP. ...., legal representative of the patient .... (species), ... (breed), ... (age), ..., (Identity code – Chip number/ or Health card/ Passport number).

Objectives of the agreement / Common objectives:

Use of the resulting data (analyzes, imagistic data, treatment, ets) for the **research programs** of the U.L.S. “Regele Mihai I” from Romania – F.M.V.

1. U.L.S. “Regele Mihai I” from Romania - Faculty of Veterinary Medicine Timisoara, engage to ensure the activities of medical-veterinary assistance, agreed by the owner / legal representative of the patient.

2. **2**. Owner/ Legal representative of the patient is agree with the veterinary care activities, established by mutual agreement and with the use of the resulting data for the research programs of the U.L.S. “Regele Mihai I” from Romania. – F.M.V.

This agreement has the character of a document which covers the entire activity of achieving the agreed common objectives.

Dean FMV, **Owner,**

………………………….. **………………..**

LS **signature**
