# Supplementary material for: Thermographic Evaluation of the Stifle Region in Dogs with a Rupture of the Cranial Cruciate Ligament
Source: Animals (Basel). 2025 Aug 7;15(15):2317. doi: 10.3390/ani15152317 (PMC12345753; doi:10.3390/ani15152317)
Supplement: Supplementary file 1 [file animals-15-02317-s001.zip › Supplementary materials/Table S1 - Mean value of temperature El1 area.pdf]

Supplementary material – Mean value of temperature El1 area

Group	Average value of temperature °C	Max value of temperature °C
Dogs with intact cranial cruciate ligament	35.52	36.15
Dogs with rupture of the cranial cruciate ligament	37.32	37.84
Differences in temperature between groups °C	1.8	1.69
