# Supplementary material for: Thermographic Evaluation of the Stifle Region in Dogs with a Rupture of the Cranial Cruciate Ligament
Source: Animals (Basel). 2025 Aug 7;15(15):2317. doi: 10.3390/ani15152317 (PMC12345753; doi:10.3390/ani15152317)
Supplement: Supplementary file 1 [file animals-15-02317-s001.zip › Supplementary materials/Table S2 - Mean value of temperature Bx1 area.pdf]

Supplementary material – Mean value of temperature Bx1 area

| Group | Average value of temperature °C | Max value of temperature °C |
|---|---|---|
| Dogs with intact cranial cruciate ligament | 35.64 | 36.22 |
| Dogs with rupture of the cranial cruciate ligament | 37.40 | 37.78 |
| Differences in temperature between groups °C | 1.76 | 1.56 |
