# Supplementary figures and images for: Thermographic Evaluation of the Stifle Region in Dogs with a Rupture of the Cranial Cruciate Ligament
Source: Animals (Basel). 2025 Aug 7;15(15):2317. doi: 10.3390/ani15152317 (PMC12345753; doi:10.3390/ani15152317)

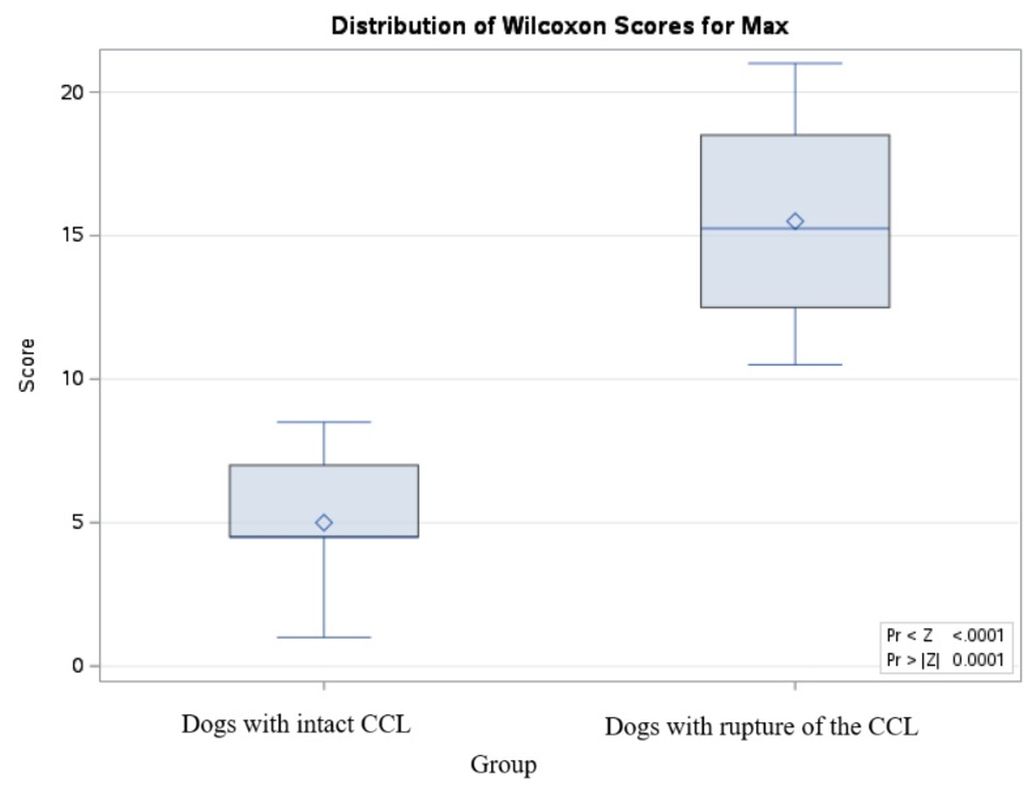

Supplement: Supplementary file 1 [file animals-15-02317-s001.zip › Supplementary materials/Figure S1 - Distribution of Wilcoxon score for Maximum in El1.jpg]

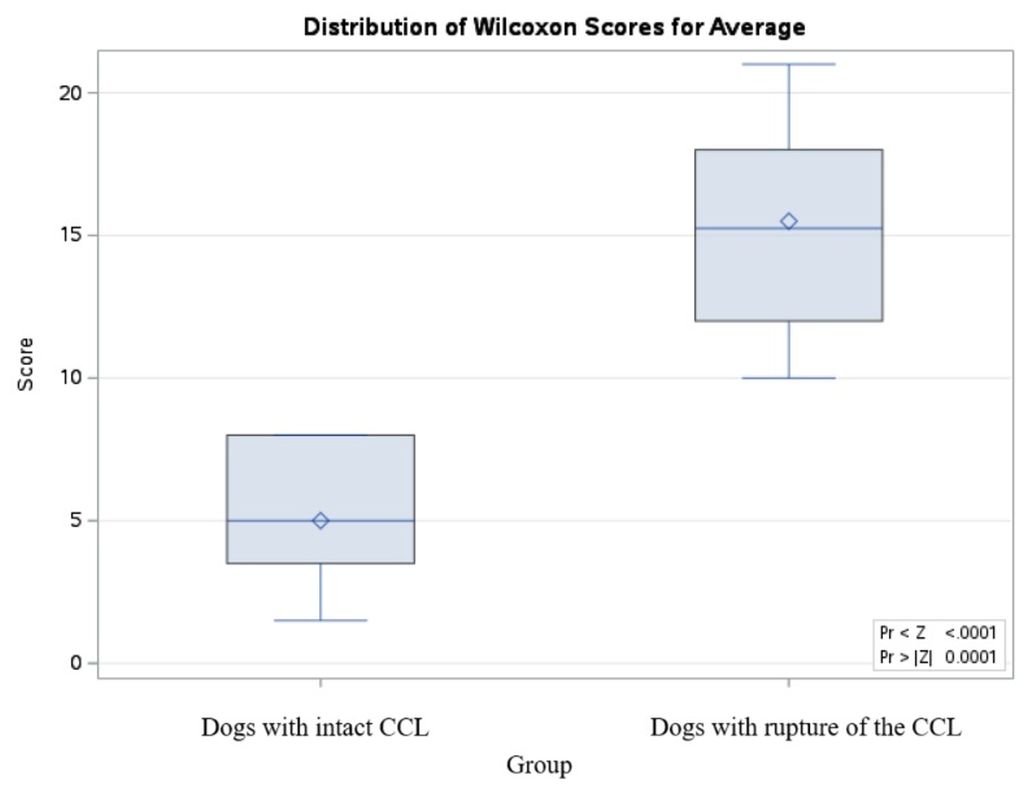

Supplement: Supplementary file 1 [file animals-15-02317-s001.zip › Supplementary materials/Figure S2 - Distribution of Wilcoxon score for Average in El1.jpg]

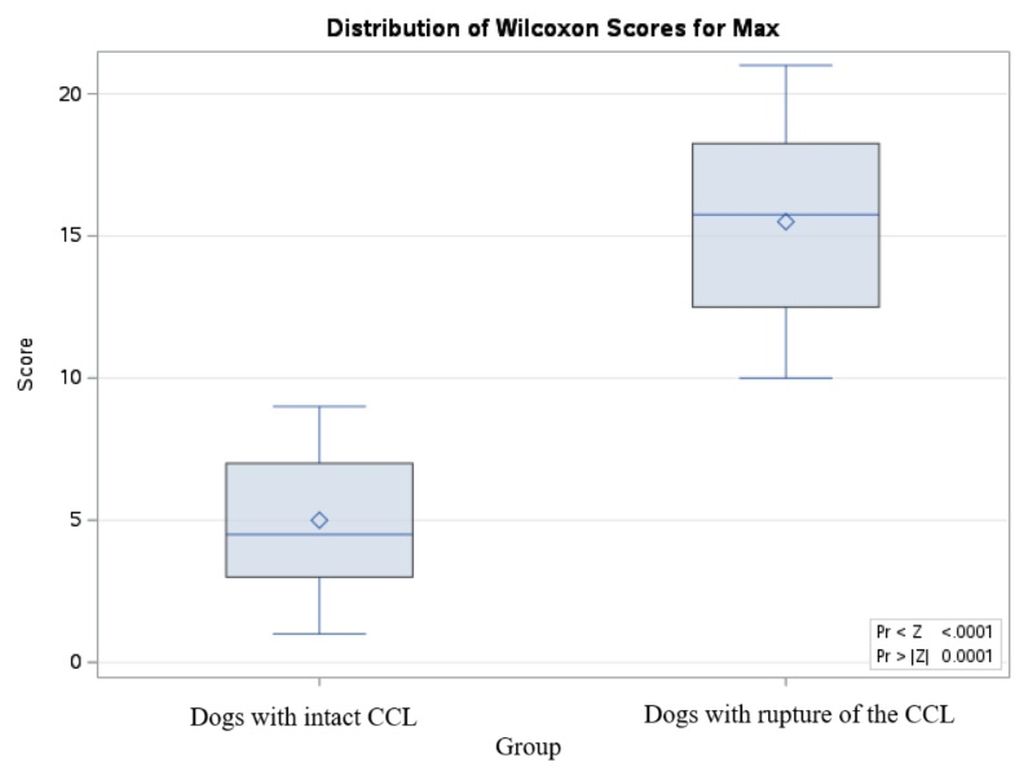

Supplement: Supplementary file 1 [file animals-15-02317-s001.zip › Supplementary materials/Figure S3 - Distribution of Wilcoxon score for Maximum in Bx1.jpg]

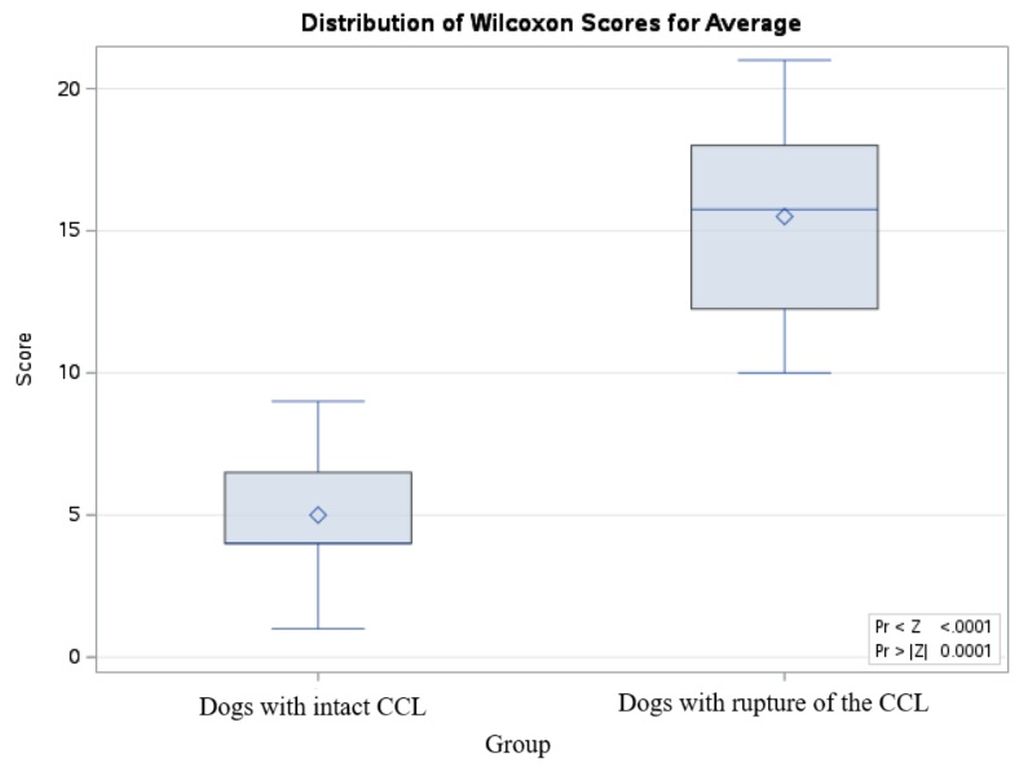

Supplement: Supplementary file 1 [file animals-15-02317-s001.zip › Supplementary materials/Figure S4 - Distribution of Wilcoxon score for Average in Bx1.jpg]
